# Levels and Health Risk of Pesticide Residues in Chinese Herbal Medicines

**DOI:** 10.3389/fphar.2021.818268

**Published:** 2022-02-01

**Authors:** Ying Wang, Yan Gou, Lei Zhang, Chun Li, Zhao Wang, Yuanxi Liu, Zhao Geng, Mingrui Shen, Lei Sun, Feng Wei, Juan Zhou, Lihong Gu, Hongyu Jin, Shuangcheng Ma

**Affiliations:** ^1^ Institute for Control of Chinese Traditional Medicine and Ethnic Medicine, National Institutes for Food and Drug Control, Beijing, China; ^2^ Sichuan Institute for Drug Control, Sichuan Testing Center of Medical Devices/NMPA Key Laboratory of Quality Evaluation of Chinese Patent Medicines, Chengdu, China; ^3^ China National Center for Food Safety Risk Assessment, Beijing, China; ^4^ Guangzhou Institute for Drug Control, NMPA Key Laboratory for Quality Evaluation of Traditional Medicine, Guangzhou, China; ^5^ Chinese Pharmacopoeia Commission, Beijing, China

**Keywords:** chinese herbal medicines, pesticide residues, exposure frequency, risk scoring, cumulative evaluation

## Abstract

In the present study, 168 pesticides in 1,017 samples of 10 Chinese herbal medicines (CHMs) were simultaneously determined by high-performance liquid (HPLC-MS/MS) and gas (GC-MS/MS) chromatography–tandem mass spectrometry. A total of 89.2% of the samples encompassed one or multiple pesticide residues, and the residue concentrations in 60.5% of samples were less than 0.02 mg kg^−1^, revealing the relatively low residue levels. The hazard quotient and hazard index methods were used to estimate the health risk for consumers. For a more accurate risk assessment, the exposure frequency and exposure duration of CHMs were involved into the exposure assessment, which was obtained from a questionnaire data of 20,917 volunteers. The results of chronic, acute, and cumulative risk assessment indicated that consumption of CHMs is unlikely to pose a health risk to consumers. Ranking the risk of detected pesticides revealed that phorate, BHC, triazophos, methidathion, terbufos, and omethoate posed the highest risk. Our results also showed that pollution of the aboveground medicinal part was more serious. Although exposure to pesticides in tested CHMs was below dangerous levels, more strict controlled management should be carried out for banned pesticides due to the high detection rate and illegal use in the actual planting practice.

**GRAPHICAL ABSTRACT:**
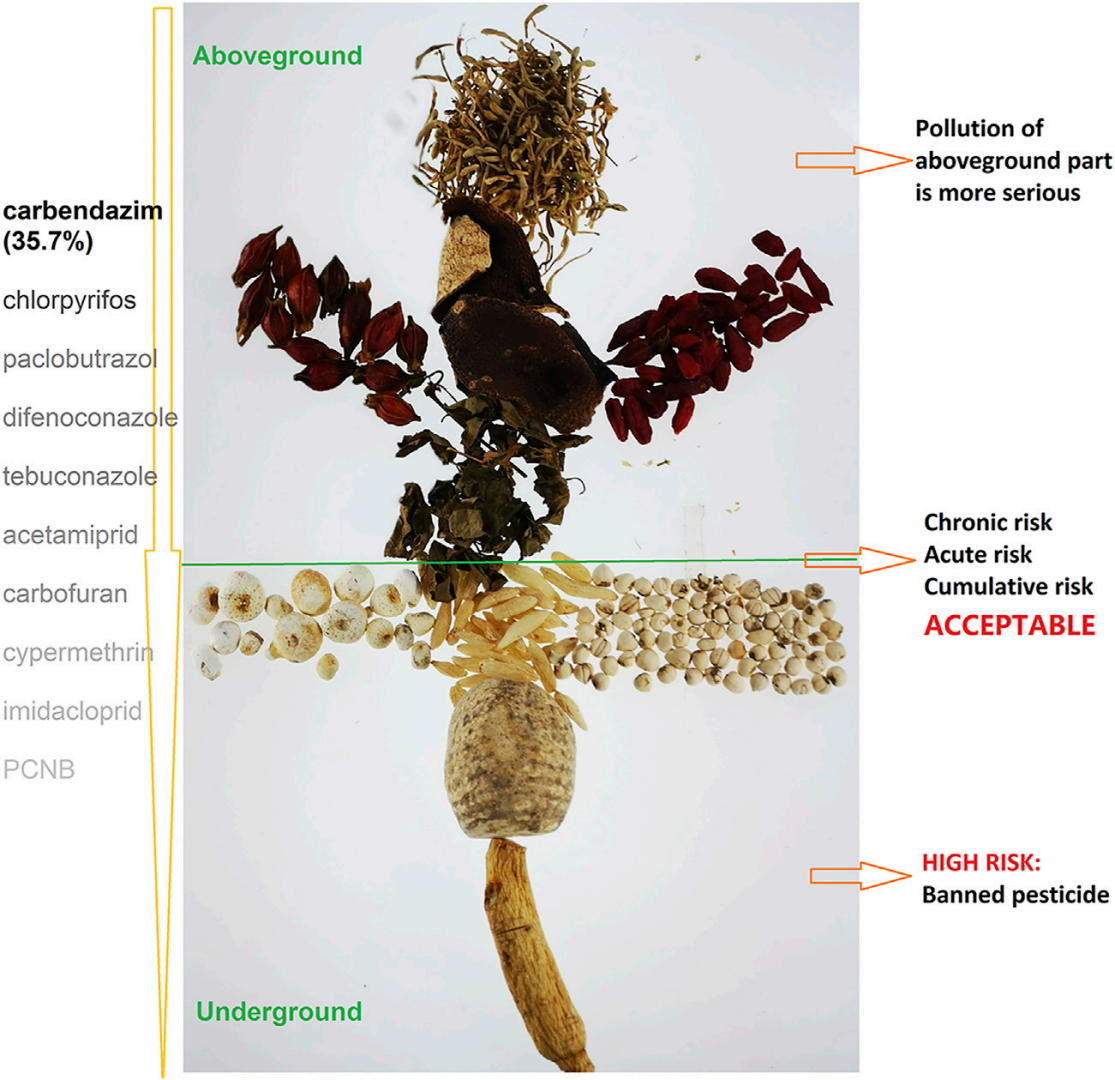


## Introduction

Recently, the efficacy of Chinese herbal medicines (CHMs) has been generally recognized by domestic and foreign markets. The World Health Organization (WHO) indicates that 75% of the worldwide population use CHMs for their fundamental medical and health care needs (Pan et al., 2014). At the same time, with the increasing global acceptance of CHMs, their use has also expanded to other sectors such as health products, food supplements (Piemontese, 2017), cosmetics (Xie et al., 2015), and food-flavoring agents (Nabavi et al., 2015).

With the rapid increase of demand for CHMs in the global market, concerns for the efficacy and safety of CHMs were raised. The efficiency of CHMs is controversial due to the lack of literature reports. Teschke *et al.* studied the literature of traditional Chinese medicines (TCMs) used in the treatment of gastrointestinal disorders. When analyzing published clinical trials, it was found that all indications lacked placebo-controlled, randomized, double-blind trials (Teschke et al., 2015). Finally, it is considered that although the use of TCM in the treatment of various diseases has a long history, high-quality test verification is lacking. Totally, 13 journals related to TCM published in China were randomly selected by Wang et al., and the reporting quality of randomized controlled trials (RCTs) in TCM journals was evaluated. The findings of this study suggested that the reporting of some important methodological components of RCTs is incomplete, and the reporting quality of these trials still needs to be improved (Wang et al., 2007). Investigations assessing the adverse effects of TCM mainly focus on liver damage, which is rarely found in a German TCM hospital that carefully analyzed TCM preparations for product quality before treatment of patients (Melchart et al., 2017). Many phytochemicals have been confirmed to be beneficial to human health, for example, exerting hepatoprotective effects (Domitrovi’c and Potocnjak, 2016), but more cases of liver injury caused by phytocompounds have been reported with increasing frequency. The risk assessment of herb-induced liver injury (HILI) for individual cases was achieved using the Roussel Uclaf Causality Assessment Method (RUCAM) (Danan Teschke, 2016) under the recommendations of the Chinese Society of Hepatology (GSH) (Yu et al., 2017). With the data provided for worldwide published 12,068 HILI cases (Teschke et al., 2020), the risk of liver injury associated with the use of CHMs was evidenced by using RUCAM to verify a causal relationship. The investigation of the causes of HILI is the focus of attention at present.

It has been reported that potential hepatotoxins in herbal medicinal products may be related to the presence of plant-originated components (phyto-hepatotoxins) as well as exogenous pollutants (non-phyto-hepatotoxins) (Quan et al., 2020). The term “phyto-hepatotoxins” refers to any potential hepatotoxic compound synthesized by medicinal plants, most of which are secondary metabolites produced to protect medicinal plants from external attacks. Recently, a total of 296 phytochemicals have been reported to have potential hepatotoxicity, of which alkaloids and terpenoids are the two major categories (He et al., 2019). For example, there have been many reports of liver injury induced by pyrrolizidine alkaloids (PAs) (Wiesner, 2021; Steinhoff, 2021b). The toxicity of plants containing certain PAs has long been recognized in grazing animals and humans. In 2013, PAs in 221 herbal teas and herbal tea samples were analyzed by the German Federal Institute for risk assessment (BfR) [BfR (German Federal Institute for Risk Assessment), 2013], and the potential contamination of weeds containing PAs (such as *Senecio* species) to medicinal plant materials is being discussed. The herbal Medicinal Products Committee (HMPC) issued a public statement on the possible contamination of herbal products with PAs in 2016. In 2020, the HMPC recommended in its new draft public statement a daily intake of 1.0 µg of pyrrolizidine alkaloids per day for adults, including contamination of herbal products in 2020. Generally, exogenous contaminants are divided into three main groups, including metals, mycotoxins, and pesticide residues (Steinhoff, 2021a). Especially, pesticides in CHMs can directly influence their safety and efficacy, while long-term exposure to pesticide residues may cause toxic chemicals to accumulate in the body. Chronic pesticide poisoning may lead to endocrine disorders, infertility, immunosuppression, carcinogenic, and teratogenic effects (Singh et al., 2018).

Due to the increasing scarcity of wild CHM resources, artificial planting has become the primary source of CHMs. Nearly half of the 600 common CHMs were artificially planted (Wei et al., 2015). The utilization of pesticides is inevitable in large-scale planting of CHMs due to the occurrence of diseases and insect pests. Pesticide residues seriously affect the quality and safety of CHMs and export trade, which is a matter of great concern for the international market (Zhang L. et al., 2012). In 2013, Greenpeace stated that pesticide residues were identified in 74% of CHM samples, and the amounts of residues in particular samples were several times the respective maximum residue limits (MRLs) established by the European Union (Greenpeace, 2013). They reached the conclusion that pesticide residues in CHMs pose health safety problems. The MRL is usually used to judge whether pesticide residues in the product meet the quality requirements. In the current (ChP Chinese Pharmacopoeia, 2020) edition (ChP Chinese Pharmacopoeia, 2020), the 33 banned pesticides were limited in plant medicinal materials; in the European pharmacopoeia 9th edition [EP (European Pharmacopiea), 2018] and the US pharmacopoeia 41st edition (USP The United States Pharmacopeia, 2018), the MRLs of 77 pesticides in herbal medicines were established. At present, the screening of pesticide residues in CHMs often involves hundreds of pesticide indicators, most of which have no MRL, making it difficult to determine whether they exceed the standard values. Moreover, the MRL is a product limit and not a safety limit (Wanwimolruk et al., 2015). Foods that contain pesticide residues beyond the recommended MRLs are not necessarily unsafe. In several instances, there is a margin between counted intake and health-based guidance values. Consumers intake level and consumption frequency are important factors affecting the conclusions of risk assessment.

As a main risk assessment method, exposure assessment is commonly used for evaluating the risk of chemicals in food and the environment. State agencies use the obtained data for making regulation policies. Short- and long-term risk assessments are commonly used in exposure assessments for acute and chronic risk analyses of CHM ingestion, respectively. Cumulative risk assessment is necessary to evaluate the accumulation of pesticides in CHMs because many herbs may be polluted by more than one pesticide, which may cause combined effects. In conjunction with dietary risk evaluation, an adequate scoring method would be beneficial for highlighting the chemical risk in food monitoring. The risk ranking scheme method, which was proposed by the British Veterinary Drug Residues Committee [VRC (The Veterinary Residues Committeee Matrix Ranking Subgroup), 2013], has been described in many studies (Nie et al., 2014; Fang et al., 2015; Li et al., 2015).

Previous risk assessment analyses have mainly been focusing on vegetables and fruits. Recently, researchers have paid more attention to systematic evaluation and risk analysis of several pesticides in Chinese herbs (Luo et al., 2021; Xiao J. et al., 2018; Xiao J. J. et al., 2018). The hazard quotient (HQ) and hazard index (HI) methods have been applied to evaluate the potential health risk of CHMs in recent research (Wu et al., 2020). However, most of these studies refer to the food model without considering the consumption characteristics of CHMs. China is the biggest producer and exporter of CHMs. Therefore, the risk assessment and pollution situation of pesticides in CHMs are a great matter of concern in China as well as worldwide. At present, Chinese state agencies have realized the importance and urgency of enhancing pesticide regulation in CHMs. The goal of this research was to explore the contamination status and perform the risk assessment of pesticide residues in CHMs in China to provide monitoring suggestions for the CHM industry. The risk of exposure to pesticides in CHMs has been ranked by utilizing a matrix ranking process. Moreover, a risk assessment model applied for the characteristics of CHMs has been explored and proposed.

## Materials and Methods

### Materials and Reagents

Pesticide standards were provided by the Ministry of Agriculture (Beijing, China), the National Institute for Food and Drug Control, and Dr. Ehrenstorfer GmbH, and all had >96% purity. The Carb/NH_2_ SPE cartridge (500 mg, 6 ml), HLB SPE cartridge (500 mg, 6 ml), and PSA (40–63 μm, 60 Å) were acquired from Agela Technologies (Tianjin, China). QuEChERS (Quick, easy, cheap, effective, rugged, and safe) silica gel dispersive purge tubes (containing 300 mg C_18_, 300 mg PSA, 90 mg GC-e, 300 mg Silica gel, and 900 mg anhydrous MgSO_4_) used for dispersive solid-phase extraction analysis were from Shimadzu (Japan). Analytical sodium chloride, glacial acetic acid, and solvents were provided by Sinopharm Chemical Reagent Co., Ltd. (Shanghai, China). HPLC grade acetonitrile and acetone were from Fisher Scientific (United States).

Individual 100 μg/ml pesticide stock solutions were prepared in toluene or acetone and stored at −20°C until analysis. Mixtures of working standard solutions at a series of concentrations were made by diluting aliquots of the stock mixture in acetonitrile.

### Sample Collection

A Total of 1,017 samples, including 127 Ginseng radix rhizome (GR), 47 Lycii fructus (LF), 98 Houttuyniae herba (HH), 125 Ophiopogonis radix (OR), 105 Alismatis rhizome (AR), 35 Citri reticulatae pericarpium (CR), 155 Chuan Bei Mu (including *Fritillaria cirrhosa,* Fritillaria unibracteata, Fritillaria przewalskii, Fritillaria delavayi, Fritillaria taipaiensis, Fritillaria unibracteata) (FC), 175 Pinelliae rhizome (PR), 105 Gardeniae fructus (GF), and 45 Lonicerae japonicae flos (LJ) specimens, were collected in major production regions in China. The samples were purchased from cultivation regions, herbal markets, decoction companies, and pharmacies, representing almost all available channels for purchasing CHMs in China. Samples were 3 kg or more and categorized by quartering. Samples were kept at −20°C until analysis.

### Sample Preparation

Method 1 (pretreatment of GR, LF, CR, and LJ samples): 5 g of the sample (powder) was accurately weighed into a 50 ml tube after homogenization; then, 3.0 g sodium chloride and 50 ml acetonitrile were added into the centrifuge tube. After shaking for 2 min, the mixture was centrifuged at 4,000 rpm for 5 min. The upper layer was moved to another 50 ml centrifuge tube. Next, 50 ml acetonitrile was added into the residue of the crude extract and vortexed for 2.0 min, after which the supernatant was combined and transferred into a round-bottom flask and evaporated at 40°C till near dryness. The resulting residue was dissolved in 10.0 ml acetonitrile. Totally, 2.0 ml extract was then loaded into a Carb/NH_2_ column, which was prepared using 5 ml of acetonitrile: toluene 3:1 (v/v). The extract solution was passed through the column at a flow rate of 1 ml min^−1^. The retained analytes were eluted with 20 ml of acetonitrile: toluene 3:1 (v/v). The collected eluate was evaporated at 40°C to near dryness. Finally, the residue was redissolved in 5.0 ml acetonitrile for analysis.

Method 2 (pretreatment of HH, OR, AR, FC, PR, and GF samples): 3 g of the homogenized sample was accurately weighed into a 50 ml centrifuge tube, and 15 ml of deionized water (containing 1% acetic acid) was added and evenly vortexed. After incubation at room temperature for 30 min, 15 ml acetonitrile was added and vortexed for 5 min and immediately cooled in an ice-water bath for 30 min. A WondaPak QuEChERS extraction package containing anhydrous magnesium sulfate (MgSO_4_, 6 g) and anhydrous CH_3_COONa (1.5 g) was added; then, the tube was vigorously vortexed for 5 min and immediately cooled in an ice-water bath for 10 min. The tube was centrifuged at 8,000 rpm for 5 min to separate the two layers. For further cleanup, 8 ml of the supernatant was transferred to a 15 ml WondaPak QuEChERS Silica gel dispersive purge tube. The mixture was vigorously vortexed for 5 min and centrifuged at 8,000 rpm for 5 min. Then, the supernatant was filtered through a 0.22 μm nylon organic filtration for analysis.

### Sample Analysis

#### UPLC-MS/MS

A Waters Acquity UPLC instrument interfaced with a XEVO Triple Quad mass spectrometry system (Waters Co., United States) was used for sample analysis. Separation was carried out on a 2.10 × 100 mm column (ACQUITY UPLC BEH C_18_ column; Waters). The mobile phase included solutions A (5 mM ammonium formate and 0.1% formic acid in water) and B (5 mM ammonium formate and 0.1% formic acid in 95% methanol); the following gradient was applied at a flow rate of 0.4 ml min^−1^: 0–0.8 min, 20% B; 0.8–11.0 min, 20–100% B; 11.0–13.0 min, 100% B; 13.0–14.0 min, 100–20% B; 14.0–18.0 min, 20% B. The injection volume was 1 μl. The drying gas flow was at 8 L/min, and the oven temperature was 30°C. Detection was performed in the multiple reaction monitoring (MRM) modes, operated in the electrospray positive/negative ionization mode (ESI^+^/ESI^−^). Instrument parameters were optimized to improve sensitivity, and the source temperature, cone voltage, desolvation gas flow, cone gas flow, and desolvation temperature were set at 150°C, 30 V, 900 L/Hr, 50 L/Hr, and 500°C, respectively.

#### GC-MS/MS

A Shimadzu gas chromatograph equipped with a tandem mass spectrometer quadrupole QP2010 (EI source) was used to perform analysis with a DB-17MS capillary column (30 m length × 0.25 mm id × 0.25 mm film thickness). The oven temperature was programmed at 60°C for 2 min, after which it was gradually increased to 150°C at a rate of 15°C/min and to 280°C at 6°C/min, held for 8 min. The inlet temperature was 250°C. The injection volume was at 1 µl in the splitless mode. The carrier gas was helium, used at a flow rate of 1.0 ml/min. The mass spectrometer was operated in the MRM mode with nitrogen as the collision gas at a flow rate of 1.5 ml/min. The temperatures of the ion source and transfer lines were 230°C and 280°C, respectively. The solvent delay was set at 6.0 min.

### Health Risk Assessment

#### Chronic and Acute Intake Risk Assessments

Oral exposures to pesticide residues in CHMs were estimated by combining the concentration data with consumption data for CHMs. Body weight data were obtained from WHO statistics [WHO (World Health Organization), 2016]. The acceptable daily intake (ADI) and acute reference dose (ARfD) were obtained from the JMPR database [WHO (World Health Organization), 2017]. The resulting dietary exposure estimate was then compared with relevant toxicological reference values (such as ADI or ARfD) for the pesticides of concern. Assessments were undertaken for chronic (long-term) or acute (short-term) exposures. The chronic hazard quotient (HQc) and the acute hazard quotient (HQa) were used to evaluate the chronic and acute dietary exposure risks, respectively. HQc and HQa were calculated according to Eq. 1, 2, respectively:

$$HQc=\frac{R\times CR\times EF\times Ed}{bw\times AT\times ADI}$$ (1)

where R is the average residue level of the pesticide in the sample (mg/kg); *CR* is the average CHM consumption (kg/day); EF is the exposure frequency, which was 90 days per year based on the previous investigation; Ed represents the exposure time, which was 20 years according to the questionnaire results; and AT is the average time, which was always equal to life expectancy, 365 days/year × 70 years; bw is the average body weight of Chinese adults (63 kg). When HQc <1, the risk was considered acceptable; at HQc >1, an unacceptable risk was considered; the higher the value, the higher the risk

$$HQa=\frac{HR\times LP}{bw\times ARfD}$$ (2)

where HR is the highest residue and LP is the large portion (kg). At HQa <1, the risk does not constitute a health threat in the short term. Conversely, when HQa is higher than 1, an unacceptable risk is considered; the higher the HQa value, the greater the acute risk exposure.

When carrying out exposure assessments, the percentage of left-detected numbers in the data set of residue concentration is vital. Treatment and calculation of these values may affect the assessment results (Ooijen et al., 2009). In this study, residue concentrations lower than LOQs were treated as 0.5 × LOQ according to WHO recommendations and seemed acceptable (GEMS/food Global Environment Monitoring System, 2016).

#### Cumulative Risk Assessment

The hazard index (HI) is a parameter used for cumulative risk assessment (Asante Duah, 1998; Li et al., 2018), which is expressed as the sum of HQc values for each pesticide in the sample according to Eq. 3. The HI measurement is clear, understandable, and directly related to the reference dose value. As a quick and simple method, the HI measurement has been used in primary cumulative risk assessment of various sample types such as air (Xiao et al., 2015), soil (Chang et al., 2014), and food (Lyulyukin et al., 2018). In this study, the HI approach was applied to evaluate the cumulative risk of pesticides in CHMs

$$HI=\sum_{i=1}^{n}HQc$$ (3)

where n is the total number of pesticides. At HI > 1, the CHMs involved should be considered a risk to the consumers; meanwhile, HI < 1 indicates that the CHMs involved are considered acceptable in the long term.

#### Risk Scoring System

Pesticide residue risk score (TS) is a mixture of toxicity and exposure scores. The toxicity score is composed of distinct values for A and B, while the exposure score encompasses four distinct scores for C, D, E, and F. TS is calculated by Eq. 4:

TS=(A+B)′(C+D+E)′F (4)

where A represents toxicity, as acquired through the Ministry of Agriculture website at the People’s Republic of China (ICAMA Institute for the Control of Agrochemicals, MOA of China, 2017); B represents potency pesticide score; C is a score for the percentage of CHMs in a diet; D represents a score for the incidence of pesticide use through planting; E represents a score for the amount of highly exposed population; F is a score for pesticide levels. The higher the mean amount of pesticide residual risk score, the higher the risk.

## Results

### Method Validation

The in-house validation data fulfilled the requirements of the European SANTE/12830/2021 Guideline (European Commission, 2020). This was carried out by the investigation of the following parameters: limit of quantitation (LOQ), precision, linearity, accuracy, and matrix effect (ME). The LOQ for each pesticide was calculated as the lowest concentration of the target compound producing a signal-to-noise ratio (S/N) of 10. The accuracy and precision of the method were assessed by recovery experiments with three replicates spiked at three levels (LOQ, 5 × LOQ and 10 × LOQ). Linearity was studied by using matrix-matched calibration. The ME was obtained by comparing the signal intensity of the standard with and without the matrix at the same concentration.

The linearity of the matrix-matched calibration curve was good for all the pesticides in related concentration ranges, with correlation coefficients (*r* ^2^) greater than 0.99. The values of LOQ were well below the MRLs in vegetables and teas established by the Ministry of Agriculture of China (GB 2763-2019, 2019). The recoveries for all detected pesticides were in the range of 65.4–118.7% (RSD≦20%), indicating that the method may meet the detection requirements. The results showed that most pesticides exhibited different ME levels in 10 kinds of herbs. Thus, a matrix-matched calibration standard solution was used for quantification to avoid the inaccuracy of quantitative caused by ME.

### Pesticide Residues Identified in CHM Samples

Among the 168 detected pesticides, 84 were detected in the 1,017 samples, including 17 banned pesticides, which suggests that some farmers were still using these pesticides; alternatively, the residues of pesticides used in previous years were still high in amounts and effective in the soil. Although banned pesticides have been detected in CHMs, according to the ChP Chinese Pharmacopiea (2020) version, only 25 batches of samples exceeded the MRLs, with an unqualified rate of 2.5%. Among them, carbendazim was the most frequently detected compound (35.7%). Carbendazim is a fungicide widely used in CHMs as well as in fruits and vegetables in China, which can effectively control many diseases caused by fungi. The frequencies of detection (%) in total samples were as follows: carbendazim (35.7%) > chlorpyrifos (34.1%) > paclobutrazol (26.7%) > difenoconazole (20.5%) > tebuconazole (18.5%) > acetamiprid (17.7%) > carbofuran (17.0%) > cypermethrin (16.0%) > imidacloprid (15.0%) > pentachloronitrobenzene (PCNB, 14.6%). The residue levels ranged from 0.001 mg kg^−1^ to 38.316 mg kg^−1^. Among them, compounds with concentrations <0.020 mg kg^−1^ were 60.5%; those with a concentration range of 0.020–0.500 mg kg^−1^ were 32.3%, and pesticides with levels >0.500 mg kg^−1^ were 7.2%, indicating that most of the pesticide residues were detected in low amounts. The detection rates and detailed concentration ranges of pesticides are provided in Supporting Information (Supplementary Material S1).

**FIGURE 1:**
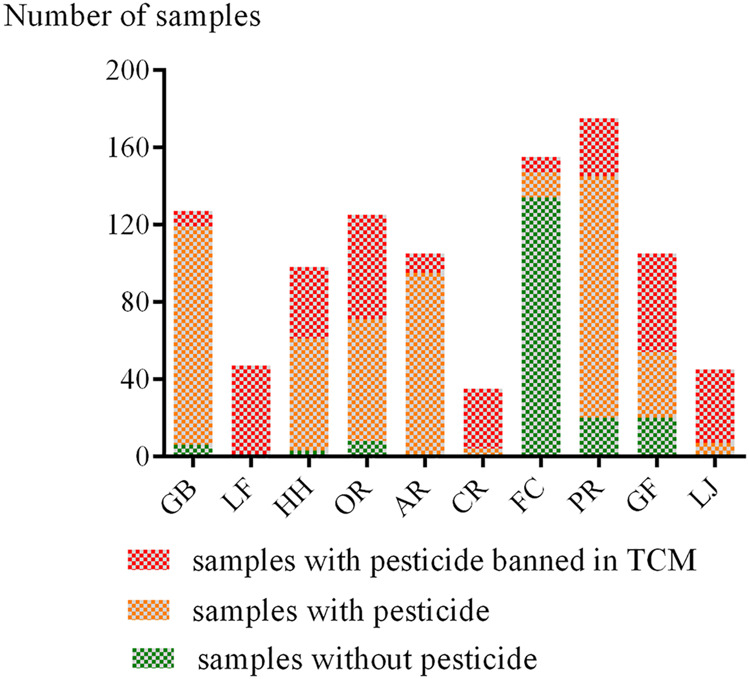
Pesticide residues in various CHMs.

Of the 1,017 analyzed samples, 110 (10.8%) were residue-free, 134 (13.2%) contained one pesticide, and 773 (76.0%) contained multiple residues. The overall rate of samples containing multi-pesticide residues was higher than the rate of samples with no or single residue. We also found that samples contaminated with more than four detectable pesticides amounted to 49.1%, indicating that many CHMs were exposed to multi-pesticide conditions. Besides, multi-pesticide residues in samples are usually a combination of one or two fungicides and one or two insecticides. The detection rate of pesticides in each Chinese herb, including the detection rate of banned pesticides is shown in Figure 1. The results showed that the detection rates of pesticides in CR, LF, LJ, and AR were the highest (up to 100%). We also found that the detection rates of banned pesticides in LF, CR, LJ, and GF were relatively high, above 50%.

### Intake Risk Assessment

#### Long-Term Consumer Exposure

The HQc values for pesticides detected in 10 CHMs are shown in Supplementary Material S2. The HQc values were 2.0 × 10^−6^–7.86 × 10^–2^ for GR, 1.0 × 10^−6^–4.61 × 10^–3^ for LF, 1.0 × 10^−6^–2.93 × 10^–2^ for HH, 4.0 × 10^−6^–1.08 × 10^–3^ for OR, 7.0 × 10^−6^–0.124 for AR, 7.0 × 10^−6^–3.02 × 10^–2^ for CR, 7.0 × 10^−6^–3.12 × 10^–3^ for FC, 4.0 × 10^−6^–3.75 × 10^–3^ for PR, 4.0 × 10^−6^–3.47 × 10^–3^ for GF, and 4.0 × 10^−6^–1.63 × 10^–2^ for LJ. Overall, for chronic exposure risk assessment, the HQc values were notably lower than 1, which indicated that long-term exposure of consumers to pesticide residues through the consumption of each of the 10 kinds of CHMs may not raise health concerns. Among the detected pesticides, the highest average value of HQc was obtained for triazophos, that is, 0.024 (mainly due to the high level of triazophos residues detected in AR). It was followed by PCNB at 0.016 and chlorpyrifos at 0.012.

#### Short-Term Consumer Exposure

Acute exposure risk assessment of 37 kinds of pesticides (including 2,4-D butylate, diflubenzuron, pyridaben, DDT, paclobutrazol, trifluralin, hexaflumuron, thiophanate-methyl, isofenphos-methyl, metalaxyl, BHC, chlorantraniliprole, permethrin, isazofos, cyprodinil, azoxystrobin, pyrimethanil, propargite, bitertanol, propamocarb hydrochloride, isocarbophos, quintozene, phoxim, omethoate, etoxazole, diethofencarb, iprodione, butralin, fludioxonil, tolclofos-methyl, metsulfuron-methyl, propoxur, prometryn, piperonyl butoxide, uniconazole, coumaphos, and fenobucarb) could not be performed since ARfD values were non-detected for these compounds or because there were no related records in the JMPR database. HQa values for the pesticides detected are provided in Supplementary Material S2. The maximum residue concentrations and consumption of CHMs were used for the determination of the worst-case scenario. The results showed that the HQa values were 3.0 × 10^−6^–1.35 × 10^–3^ for GR, 2.9 × 10^−5^–1.70 × 10^–2^ for LF, 6.0 × 10^−6^–0.179 for HH, 3.0 × 10^−6^–3.33 × 10^–3^ for OR, 3.0 × 10^−6^–1.125 for AR, 3.3 × 10^−5^–0.239 for CR, 8.0 × 10^−6^–8.33 × 10^–3^ for FC, 1.6 × 10^−5^–7.18 × 10^–5^ for PR, 2.0 × 10^−6^–6.29 × 10^–2^ for GF, and 3.0 × 10^−6^–3.83 × 10^–2^ for LJ. Except for triazophos with a HQa in AR of 1.12, the remaining pesticides had HQa values far below 1 and within the acceptable levels.

#### Risk Scoring for the Detected Pesticides in CHMs

**FIGURE 2:**
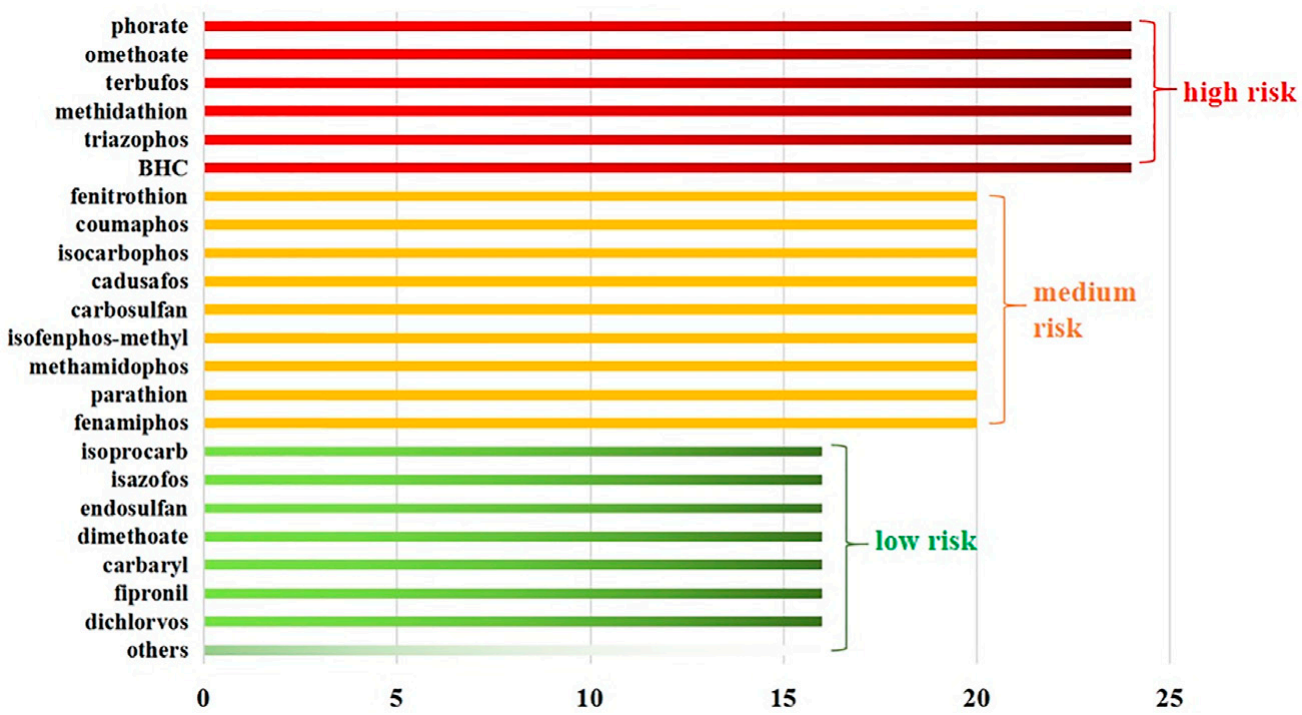
Risk scoring of 84 detected pesticides in CHMs.

A comprehensive analysis revealed that the 84 detected pesticides could be classified into three categories according to the total score determined by the matrix ranking scheme (Figure 2). Six pesticides (7.1%) had a total score of or above 24 and were considered to potentially pose a high risk in this study. Their shared feature was high toxicity, while some could induce carcinogenicity as well as reproductive and developmental toxicity. Based on the total score, 10.7 and 82.1% of the pesticides were classified into the medium-risk and low-risk groups, respectively. The calculation process of the score has been provided in the Supporting Information (Supplementary Material S3).

#### Cumulative Dietary Risk Assessment

As described in 3.2, there were 773 samples (76.0%) with multiple pesticide residues. The human body acts as a final accumulator of chemical pollutants, which can lead to health problems (Graillot et al., 2012). When two or more chemicals or other substances cause common toxicity through the same or similar main biochemical event sequence, a common toxicity mechanism can be determined. Therefore, it is necessary to analyze the cumulative risk of pesticide residues in CHMs.

We calculated the HI values for 10 CHMs and found 0.093 for GR, 0.022 for LF, 0.069 for HH, 0.004 for OR, 0.228 for AR, 0.165 for CR, 0.005 for FC, 0.006 for PR, 0.012 for GF, and 0.044 for LJ. These results indicated that the cumulative intake of multiple pesticides through the consumption of CHMs was not likely to pose a health risk to consumers.

## Discussion

### Selection of CHMs and Pesticide Indexes

A total of 10 herbs that are widely used and sold on the market were selected in this study. These 10 herbs not only have therapeutic effects but also can be used as health products in daily life. They are very representative of CHMs: GR and OR are root medicinal materials; AR, FC, and PR are stem medicinal materials; LF and GF are fruit medicinal materials; LJ is a flower medicinal material; HH is a whole plant; and CR is a fruit peel. In addition, the genuine areas of the 10 selected herbs are distributed all over the country; the medicinal parts of the selected herbs are from the underground part to the aboveground part, and the growing environments vary from a low altitude to a high altitude. The representativeness of samples has a great influence on the accuracy of risk assessment results. The varieties of CHMs selected in this study have a good reference value for reflecting the pesticide residues of CHMs in China.

In the early stages of this study, a large number of field investigations were conducted on these 10 medicinal herbs, the pests, and common diseases, and the used pesticides are well studied and understood. At the same time, referring to the relevant guidance of the Ministry of Agriculture on high-toxicity and high-residue pesticides, 168 pesticides were finally selected as detection indexes. The 168 tested pesticide indicators are very representative and mainly include most pesticides that are banned in China as well as the pesticide varieties commonly used in the cultivation of CHMs.

### Risk Assessment of Pesticides in Different Medicinal Positions

**FIGURE 3:**
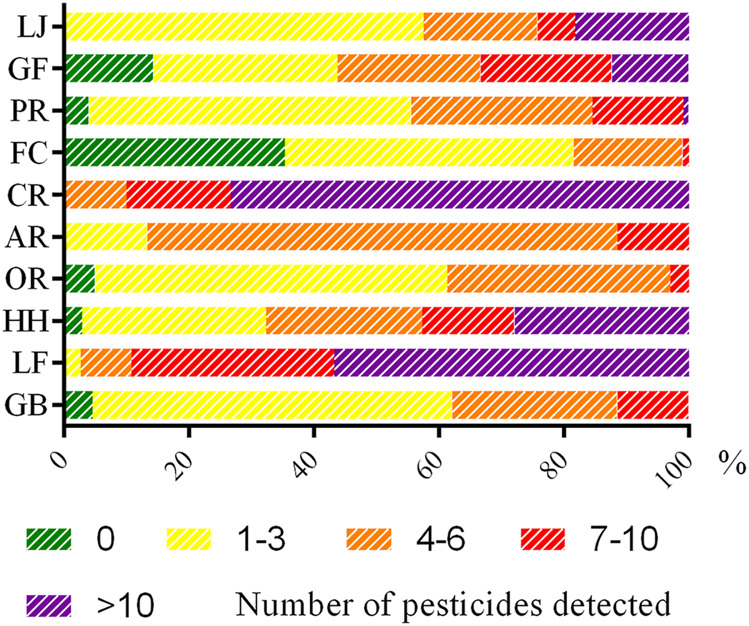
Distribution of pesticides in various CHMs.

In this study, the pesticide pollution of different medicinal parts substantially varied. Pesticide residues were more frequently detected in the whole grass or some aboveground herbs (such as flowers and fruits) compared with other medicinal parts. CHMs are summarized in Figure 3. The results revealed the presence of four or more pesticides in samples: CR accounted for 100%, and LF accounted for 97%; meanwhile, GR and OR accounted for only 38 and 36%, respectively. A total of 10 or more pesticides were detected in samples, with CR accounting for 73%, LF accounting for 57%, and GR and OR accounting for 0%. In particular, 22 pesticides were detected in one batch of CR samples. This may be due to the large contact area of this medicinal material when spraying pesticides in the planting process. It also suggests that certain CHM samples, including flowers and fruits, tend to be susceptible to a variety of diseases and insect pests in the planting process, which is why growers need to apply different pesticide varieties.

There were also differences in the types of pesticides detected in various medicinal parts. We divided 10 medicinal materials into two categories: the rhizome and aerial part. Then, the distribution of pesticides with a detection rate of more than 5% in both herb categories was analyzed. The results indicated that the detection rate of insecticides in the aboveground parts of herbs was relatively high, for example, carbofuran, fenpropathrin, cypermethrin, fenvalerate, etc., while root and rhizome herbs were more likely to be polluted by organochlorine and plant growth regulators, which might be due to the long-term contact with soil in the cultivation process. The pesticides in the soil absorbed by plants mainly originate from the pesticides sprayed on this soil before, rather than the horizontal transfer process, which is defined as the component release by donor plants and intake by the roots of acceptor plants (Selmar et al., 2019). Recent studies have outlined that alkaloids, including PAs, are transferred from living donor plants to the nearby plants.

### Uncertainty Analysis

In most instances, the median residue value of pesticides is lower than the mean value in CHMs. In most international institutions, the average value is preferred in the assessment of chronic exposure of most pollutants [EFSA (European Food Safety Authority), 2009; FAO (Food and Agriculture Organization)/WHO (World Health Organization), 2011]. If the median is selected as average consumption, the possibility of individuals in the population contacting high-pollution food in their lifetime might be negligible due to median values not being affected by high-pollution samples. Moreover, the human body should have the opportunity to take in food at each pollution level during the lifetime, so the average value is closer to the average levels in relation to the human body’s lifetime intake of pollutants. In this study, the average concentration was used because it is suitable and conservative for estimating the worst-case scenario.

How to accurately evaluate the acute intake risk of pesticide residues remains an unresolved issue. Studies have used the P99.5 for acute risk evaluation (Zhang Z. H. et al., 2012), while others used a P97.5 instead (Zhao et al., 2013). In this study, the maximum residue concentration was used for HQa assessment. Different results would be obtained in two case scenarios based on the HR value at the maximum point or at the P97.5 in the short-term risk assessment. The acute exposure risk of triazophos in AR was 1.12, which indicated that the acute risk from pesticide exposure via AR consumption was unacceptable in the short term. However, the acute exposure risk of triazophos in AR was notably decreased to an acceptable value of 0.87 when a P97.5 value was used for the calculation.

The daily consumption ranges of these 10 herbs were described by the Pharmacopoeia of the People’s Republic of China. When enough accurate consumption data are available, risk assessment results are more accurate. The daily consumption ranges of these 10 herbs were 5–25 g. Thus, 0.015 kg/d (median value) and 0.025 kg/d (max value) were used as the average and maximum consumption levels, respectively. In risk assessment of pesticides in CHMs, consumers are considered to take CHMs during the lifetime in the calculation of chronic exposure assessment (Luo et al., 2021; Xiao J. et al., 2018). It is more conservative because it is not taking the frequency of CHM consumption into consideration. To obtain more accurate risk assessment results, realistic consumption of CHMs should be considered in exposure assessment. From 2017 to 2018, a questionnaire survey about the consumption of CHMs was conducted in two cities (Beijing and Chongqing) and nine provinces in China. A total of 20,917 volunteers (11,497 women and 9,420 men) participated in the survey. Among these volunteers, the age range was 18–70 years. Among these volunteers, 72.63% were 18–44 years old, 19.51% were 45–59 years old, 7% were 7 years old, and 7.86% were over 60 years old. There are 11,358 urban residents and 9,559 rural residents who participated in the survey. According to the questionnaire data, the duration of P95 CHM intake was 90 days per year, and the exposure time was 20 years (Zuo et al., 2019). In the present study, the frequency of CHM consumption and exposure duration were used in chronic exposure assessment to make the results closer to the real situation.

It is important to note that the dietary risk in this study came from the consumption of raw products; thus, the processing factor (PF) was defaulted to 1. It would led to higher or lower estimations of the risk of pesticides in CHMs without consideration of PF. There are many studies on PF in food health risk assessment (Yigit and Velioglu, 2020). However, the processing of CHMs is different from that of food, for example, Chinese patent medicines containing ginseng are mostly processed by water decoction or 75% ethanol extraction. The transfer rate of pesticides may vary greatly under different processing conditions. According to our previous study (Wang et al., 2019), the transfer rate of PCNB in ginseng after water decoction is less than 1%, while the transfer rate of PCNB is as high as 95% with 75% ethanol extraction. The PFs of pesticides in CHMs will be further studied and included into the exposure assessment model to improve the accuracy of the assessment.

### Guidelines and Pesticide Use Advice

In this study, the HI method was used in the chronic, acute, and cumulative risk assessment of pesticides in CHMs. Risk assessment studies examining pesticides in CHMs refer to the food model. However, in view of the significant differences between CHMs and food, it is very important to explore crucial parameters for pesticide risk assessment in CHMs. The National Medical Products Administration (NMPA) has been committed to such research. Through a large number of questionnaires associated with CHM consumption characteristics, the risk assessment model for pesticides in CHMs was proposed. In China, we established a health risk assessment model for pesticides in CHMs for the first time, which is a realistic and refined model applicable to CHMs. To better monitor the safety of CHMs, the “Guidelines for risk assessment of exogenous harmful residues in Chinese herbal medicine” were proposed by our study and published in the 2020 edition of the Chinese Pharmacopoeia. Meanwhile, the guidelines have been submitted to the International Regulatory Cooperation for Herbal Medicines (IRCH) of the WHO. The guidelines we proposed have been applied to assess the risk of heavy metals in CHMs (Zuo et al., 2020). These guidelines are of great significance in assessing the risk of pesticide residues in CHMs and provide data support for the formulation of pesticide regulatory policies in CHMs.

The risk ranking scheme method considers toxicity as a risk of greater importance; that is, pesticide toxicity has a leading role in the score. In addition, another advantage is that the risk score can also be calculated for pesticides that lack ADI values or have carcinogenic effects. Of the high-risk and medium-risk pesticides examined in this study, 80% were banned by the Ministry of agriculture of China. Although exposure to pesticide residues in most tested CHMs was below dangerous levels, the present results showed that banned pesticides with relatively high detection rates may pose the highest risk, indicating that more strict control management should be carried out for banned pesticides. In addition, while planting traditional Chinese medicine herbs, the usage history and residual background of soil should be investigated in advance to prevent the CHMs from being polluted.

## Conclusion

We investigated pesticide residues in 1,017 samples of 10 CHMs and assessed potential health risk to inhabitants. The results of health risk assessment, including chronic, acute, and cumulative risk assessment, indicated that the consumption of CHMs is unlikely to pose a health risk to consumers. The risk ranking score obtained in this study showed that phorate, BHC, triazophos, methidathion, terbufos, and omethoate, three of which are prohibited in CHM planting in China, pose a relatively high risk. Consequently, more strict supervision of banned pesticides is essential to ensure the safety of CHMs. We also found that the pesticide pollution of different medicinal parts substantially varied. Pesticide residues were more frequently detected in the whole grass or some aboveground herbs (such as flowers and fruits) compared with other medicinal parts. Moreover, the detection rate of insecticides in the aboveground parts of herbs was relatively high, while root and rhizome herbs were more likely to be polluted by organochlorine and plant growth regulators. Furthermore, a health risk assessment model for pesticide residues in CHMs was established in this study. The proposed model involved the realistic exposure frequency and exposure duration of CHMs, which makes the health risk of pesticides in CHMs more scientific and accurate.

## Data Availability

The original contributions presented in the study are included in the article/Supplementary Material, further inquiries can be directed to the corresponding authors.
