# Supplementary material for: Levels and Health Risk of Pesticide Residues in Chinese Herbal Medicines
Source: Front Pharmacol. 2022 Feb 1;12:818268. doi: 10.3389/fphar.2021.818268 (PMC8844025; doi:10.3389/fphar.2021.818268)
Supplement: Supplementary file 2 [file Table1.doc]

Table 1 Detection rate of pesticides and their residue levels in 10 CHMs

| **Pesticide** | **GR** | | **LF** | | **HH** | | **OR** | | **AR** | | **CR** | | **FC** | | **PR** | | **GF** | | **LJ** | | **Total detection rate%** |
| --- | --- | --- | --- | --- | --- | --- | --- | --- | --- | --- | --- | --- | --- | --- | --- | --- | --- | --- | --- | --- | --- |
|  | Detection rate% | Residue concentration (mg/kg) | Detection rate% | Residue concentration (mg/kg) | Detection rate% | Residue concentration (mg/kg) | Detection rate% | Residue concentration (mg/kg) | Detection rate% | Residue concentration (mg/kg) | Detection rate% | Residue concentration (mg/kg) | Detection rate% | Residue concentration (mg/kg) | Detection rate% | Residue concentration (mg/kg) | Detection rate% | Residue concentration (mg/kg) | Detection rate% | Residue concentration (mg/kg) |  |
| carbendazim | 12.5 | 0.008-0.019 | 100.0 | 0.002-0.817 | 88.2 | 0.001-17.20 | 50.4 | 0.001-0.262 |  |  | 66.7 | 0.003-1.124 |  |  | 72.6 | 0.001-0.242 |  |  |  |  | 35.7 |
| chlorpyrifos | 48.8 | 0.005-0.178 | 43.2 | 0.008-0.141 | 17.7 | 0.021-2.120 | 6.4 | 0.020-0.061 | 100.0 | 0.050-7.510 | 83.3 | 0.013-0.373 | 16.9 | 0.021-0.082 |  |  | 41.0 | 0.004-0.115 | 78.6 | 0.028-0.242 | 34.1 |
| paclobutrazol |  |  |  |  | 10.3 | 0.004-1.041 | 87.2 | 0.005-2.141 | 99.1 | 0.010-0.372 | 36.7 | 0.005-0.067 |  |  | 20.3 | 0.0005-0.025 |  |  |  |  | 26.7 |
| difenoconazole | 42.5 | 0.010-0.135 | 86.5 | 0.001-0.242 | 35.3 | 0.001-0.377 | 6.4 | 0.001-0.032 |  |  | 86.7 | 0.003-0.034 |  |  |  |  | 21.0 | 0.001-0.015 | 42.9 | 0.030-0.248 | 20.5 |
| tebuconazole | 15.0 | 0.005-0.054 | 62.2 | 0.004-0.312 | 22.1 | 0.001-0.202 | 7.2 | 0.001-0.121 | 62.9 | 0.001-0.081 | 56.7 | 0.026-0.806 |  |  |  |  | 16.2 | 0.001-0.015 | 14.3 | 0.019-0.028 | 18.5 |
| acetamiprid |  |  | 100.0 | 0.005-0.345 | 33.8 | 0.0002-3.61 |  |  |  |  | 60.0 | 0.005-0.665 |  |  | 24.7 | 0.001-0.050 | 34.3 | 0.001-0.025 |  |  | 17.7 |
| carbosulfan |  |  | 97.3 | 0.001-0.214 | 35.3 | 0.0003-0.07 | 10.4 | 0.001-0.012 | 4.8 | 0.001-0.020 | 70.0 | 0.002-0.008 |  |  | 9.7 | 0.001-0.035 | 21.9 | 0.001-0.003 | 21.4 | 0.006-0.013 | 17.0 |
| cypermethrins |  |  | 67.6 | 0.084-1.418 | 4.4 | 0.310-2.804 |  |  |  |  | 73.3 | 0.021-0.192 |  |  |  |  | 56.2 | 0.006-0.300 | 92.9 | 0.189-0.890 | 16.0 |
| imidacloprid |  |  | 94.6 | 0.002-0.264 |  |  | 1.0 | 0.003 |  |  | 36.7 | 0.006-0.174 | 5.1 | 0.030-0.180 | 36.7 | 0.001-0.022 | 21.0 | 0.001-0.015 |  |  | 15.0 |
| quintozene | 77.8 | 0.005-3.830 |  |  |  |  |  |  |  |  |  |  | 4.1 | 0.010-0.025 | 24.7 | 0.001-0.051 |  |  |  |  | 14.6 |
| pyridaben |  |  | 67.6 | 0.003-0.099 | 33.8 | 0.001-2.402 |  |  |  |  | 50.0 | 0.009-4.817 |  |  |  |  | 18.1 | 0.0003-0.003 | 78.6 | 0.026-0.222 | 13.4 |
| cyhalothrin |  |  | 51.4 | 0.014-0.126 | 8.8 | 0.036-3.232 |  |  |  |  | 73.3 | 0.010-0.039 |  |  |  |  | 21.9 | 0.015-0.132 | 92.9 | 0.293-1.838 | 12.1 |
| triazophos |  |  |  |  |  |  |  |  | 66.7 | 0.130-2.711 | 33.3 | 0.037-1.537 |  |  |  |  | 30.5 | 0.004-0.15 |  |  | 11.2 |
| fenpropathrin |  |  | 24.3 | 0.012-0.045 | 17.7 | 0.02-1.114 |  |  |  |  | 53.3 | 0.016-1.708 | 1.5 | 0.110-0.235 |  |  | 39.1 | 0.002-0.305 | 35.7 | 0.008-0.029 | 10.5 |
| Thiophanate  -methyl |  |  | 81.1 | 0.003-1.296 | 8.8 | 0.001-4.508 | 18.4 | 0.0002-0.27 |  |  |  |  |  |  | 19.0 | 0.004-0.060 |  |  |  |  | 10.1 |
| procymidone | 30.0 | 0.010-0.324 | 5.4 |  | 16.2 | 0.026-1.820 | 5.0 | 0.002-0.022 | 35.2 | 0.010-0.120 |  |  |  |  |  |  |  |  |  |  | 9.8 |
| chlorantraniliprole |  |  | 10.8 | 0.008-0.046 | 23.5 | 0.001-0.204 | 7.9 |  |  |  |  |  |  |  | 7.5 | 0.001-0.012 | 33.3 | 0.001-0.025 |  |  | 8.5 |
| fenvalerate |  |  | 75.7 | 0.021-0.649 | 2.9 | 0.110-0.905 |  |  |  |  | 3.3 | 0.473-1.247 |  |  |  |  |  |  | 92.9 | 0.041-0.578 | 8.0 |
| propargite |  |  | 56.8 | 0.046-1.817 | 17.7 | 0.012-0.605 |  |  |  |  | 93.3 | 0.003-0.062 |  |  |  |  |  |  |  |  | 7.5 |
| propiconazole | 27.5 | 0.01-0.167 |  |  |  |  | 4.0 | 0.001-0.006 | 20.0 | 0.091-0.305 | 13.3 | 0.020-3.340 |  |  |  |  |  |  |  |  | 6.4 |
| iprodione | 50.0 | 0.005-0.115 |  |  |  |  |  |  |  |  |  |  |  |  |  |  |  |  |  |  | 6.2 |
| phorate | 2.5 | 0.003-0.007 |  |  |  |  | 20.8 | 0.001-0.002 | 4.8 | 0.070-0.082 | 40.0 | 0.022-0.017 |  |  | 7.5 | 0.001-0.025 |  |  |  |  | 6.0 |
| dimethomorph | 10.0 | 0.003-0.005 |  |  |  |  | 4.0 |  | 16.2 | 0.003-0.064 |  |  |  |  | 5.8 | 0.001-0.002 |  |  | 7.1 | 0.005-0.074 | 4.7 |
| azoxystrobin | 28.8 | 0.005-0.036 | 16.2 | 0.008-0.076 |  |  |  |  |  |  |  |  |  |  |  |  |  |  |  |  | 4.3 |
| phoxim | 25.2 | 0.005-0.072 |  |  |  |  |  |  |  |  | 33.3 | 0.007-0.198 |  |  |  |  |  |  |  |  | 4.3 |
| permethrin |  |  |  |  | 5.9 | 0.021-0.242 |  |  |  |  |  |  | 1.5 | 0.141-0.17 2 |  |  |  |  | 78.6 | 0.014-0.266 | 4.3 |
| hexaconazole |  |  | 10.8 | 0.009-0.027 | 5.9 | 0.003-0.108 |  |  |  |  | 53.3 | 0.008-0.117 |  |  |  |  |  |  | 28.6 | 0.030-0.108 | 4.2 |
| pyraclostrobin | 13.8 | 0.003-0.012 |  |  |  |  | 19.8 | 0.003-0.311 |  |  |  |  |  |  |  |  |  |  |  |  | 4.2 |
| fipronil |  |  | 13.5 | 0.005-0.011 |  |  |  |  |  |  | 13.3 | 0.005-0.018 |  |  |  |  |  |  | 64.3 | 0.007-0.134 | 3.9 |
| endosulfan |  |  |  |  |  |  | 1.0 |  |  |  |  |  |  |  |  |  | 26.7 | 0.001-0.905 | 14.3 | 0.006-0.016 | 3.5 |
| prochloraz |  |  | 2.7 | 0.015-0.030 |  |  | 2.0 |  |  |  | 90.0 | 0.010-0.205 |  |  |  |  |  |  |  |  | 3.5 |
| propamocarb hydrochloride | 27.5 | 0.003-0.007 |  |  |  |  |  |  |  |  |  |  |  |  |  |  |  |  |  |  | 3.4 |
| cyfluthrin |  |  |  |  | 2.9 | 0.302-3.825 |  |  |  |  | 20.0 | 0.006-0.798 |  |  |  |  |  |  | 50.0 | 0.014-0.069 | 3.2 |
| uniconazole |  |  |  |  |  |  | 24.8 | 0.005-0.032 |  |  |  |  |  |  |  |  |  |  |  |  | 3.0 |
| myclobutanil | 5.1 | 0.003-0.007 | 10.8 | 0.003-0.026 |  |  |  |  |  |  |  |  |  |  |  |  |  |  | 42.9 | 0.011-0.063 | 3.0 |
| propoxur |  |  |  |  |  |  | 1.0 |  |  |  |  |  |  |  | 15.9 | 0.001-0.012 |  |  |  |  | 2.9 |
| bifenthrin |  |  |  |  | 2.9 | 0.033-0.904 |  |  |  |  |  |  |  |  |  |  | 12.4 | 0.002-0.03 | 28.6 | 0.020-0.078 | 2.8 |
| profenofos |  |  |  |  | 5.9 | 0.105-0.401 |  |  |  |  | 53.3 | 0.034-6.449 |  |  |  |  |  |  | 7.1 | 0.003-0.006 | 2.7 |
| cyprodinil | 20.2 | 0.005-0.047 |  |  |  |  |  |  |  |  |  |  |  |  |  |  |  |  |  |  | 2.5 |
| flusilazole | 11.3 | 0.005-0.019 | 8.1 | 0.001-0.01 |  |  |  |  |  |  |  |  |  |  |  |  |  |  | 14.3 | 0.009-0.016 | 2.4 |
| etoxazole |  |  |  |  |  |  |  |  |  |  | 66.7 | 0.002-1.078 |  |  |  |  |  |  |  |  | 2.3 |
| hexaflumuron |  |  |  |  |  |  |  |  |  |  |  |  |  |  |  |  | 21.9 | 0.005-0.042 |  |  | 2.3 |
| diflubenzuron |  |  | 5.4 | 0.008-0.009 |  |  |  |  |  |  | 56.7 | 0.007-0.036 |  |  |  |  |  |  |  |  | 2.2 |
| methidathion |  |  |  |  |  |  |  |  |  |  | 63.3 | 0.005-0.008 |  |  |  |  |  |  |  |  | 2.2 |
| buprofezin |  |  |  |  |  |  |  |  |  |  | 56.7 | 0.004-0.097 |  |  |  |  |  |  |  |  | 2.0 |
| omethoate |  |  |  |  |  |  |  |  |  |  |  |  |  |  |  |  |  |  | 42.9 | 0.004-0.590 | 1.9 |
| etofenprox |  |  |  |  |  |  |  |  |  |  |  |  | 12.3 | 0.004-0.025 |  |  |  |  |  |  | 1.9 |
| bitertanol |  |  |  |  | 11.8 | 0.128-3.02 |  |  |  |  | 20.0 | 0.003-0.013 |  |  |  |  |  |  |  |  | 1.8 |
| isocarbophos |  |  |  |  | 2.9 | 11.23-12.51 |  |  |  |  | 43.3 | 0.012-0.448 |  |  |  |  |  |  |  |  | 1.8 |
| Imazalil |  |  |  |  |  |  |  |  |  |  | 43.3 | 0.035-0.048 |  |  |  |  |  |  |  |  | 1.5 |
| terbufos |  |  |  |  |  |  | 11.9 |  |  |  |  |  |  |  |  |  |  |  |  |  | 1.5 |
| pyrimethanil | 10 | 0.005-0.032 |  |  |  |  |  |  |  |  |  |  |  |  |  |  |  |  |  |  | 1.2 |
| metalaxyl | 10 | 0.005-0.013 |  |  |  |  |  |  |  |  |  |  |  |  |  |  |  |  |  |  | 1.2 |
| diethofencarb | 10 | 0.002-0.005 |  |  |  |  |  |  |  |  |  |  |  |  |  |  |  |  |  |  | 1.2 |
| Isofenphos  -methyl |  |  |  |  |  |  |  |  |  |  | 3.3 | 0.014-0.712 | 1.2 | 0.050-0.062 |  |  |  |  | 21.4 | 0.006-0.037 | 1.2 |
| BHC | 3.8 | 0.005-0.171 |  |  |  |  |  |  |  |  |  |  | 4.1 | 0.010-0.072 |  |  |  |  |  |  | 1.1 |
| butralin |  |  |  |  |  |  |  |  |  |  |  |  |  |  |  |  |  |  | 21.4 | 0.023-0.051 | 0.9 |
| parathion |  |  |  |  |  |  |  |  |  |  |  |  |  |  |  |  |  |  | 21.4 | 0.016-0.069 | 0.9 |
| triadimefon |  |  |  |  |  |  |  |  |  |  |  |  |  |  |  |  |  |  | 21.4 | 0.003-0.027 | 0.9 |
| fenpyroximate |  |  | 5.4 | 0.005-0.013 |  |  |  |  |  |  | 20.0 | 0.007-0.157 |  |  |  |  |  |  |  |  | 0.9 |
| 2,4-D butylate | 1.3 | 0.003-0.012 | 5.4 | 0.011-0.023 |  |  |  |  |  |  | 13.3 | 0.020-0.424 |  |  |  |  |  |  |  |  | 0.9 |
| piperonyl butoxide |  |  |  |  |  |  | 4.0 |  |  |  | 6.7 | 0.003-0.011 |  |  |  |  |  |  |  |  | 0.7 |
| DDT |  |  |  |  |  |  | 4.0 | 0.003-0.17 |  |  |  |  | 1.2 | 0.008 |  |  |  |  |  |  | 0.7 |
| trifluralin |  |  |  |  |  |  |  |  |  |  |  |  |  |  |  |  |  |  | 14.3 | 0.006-0.012 | 0.6 |
| carbaryl |  |  |  |  |  |  |  |  |  |  |  |  |  |  |  |  |  |  | 14.3 | 0.040-0.049 | 0.6 |
| chlorfenapyr |  |  |  |  |  |  |  |  |  |  |  |  |  |  |  |  |  |  | 14.3 | 0.048-0.102 | 0.6 |
| isazofos |  |  |  |  |  |  |  |  |  |  | 16.7 | 0.003-0.051 |  |  |  |  |  |  |  |  | 0.6 |
| malathion |  |  |  |  |  |  |  |  |  |  | 16.7 | 0.028-0.573 |  |  |  |  |  |  |  |  | 0.6 |
| dicofol |  |  |  |  |  |  |  |  |  |  | 6.7 | 0.024-1.411 |  |  |  |  |  |  | 7.1 | 0.002-0.007 | 0.5 |
| prometryn |  |  |  |  |  |  | 4.0 |  |  |  |  |  |  |  |  |  |  |  |  |  | 0.5 |
| fludioxonil | 3.8 | 0.003-0.03 |  |  |  |  |  |  |  |  |  |  |  |  |  |  |  |  |  |  | 0.5 |
| cadusafos |  |  |  |  |  |  |  |  |  |  | 10.0 | 0.005-0.017 |  |  |  |  |  |  |  |  | 0.3 |
| coumaphos |  |  |  |  |  |  |  |  |  |  | 10.0 | 0.005-0.007 |  |  |  |  |  |  |  |  | 0.3 |
| acetochlor |  |  |  |  |  |  |  |  |  |  |  |  |  |  |  |  |  |  | 7.1 | 0.006-0.041 | 0.3 |
| methamidophos |  |  |  |  |  |  |  |  |  |  |  |  |  |  |  |  |  |  | 7.1 | 0.010-0.040 | 0.3 |
| dichlorvos |  |  |  |  |  |  |  |  |  |  |  |  |  |  |  |  |  |  | 7.1 | 0.012-0.386 | 0.3 |
| acephate |  |  |  |  |  |  |  |  |  |  |  |  |  |  |  |  |  |  | 7.1 | 0.010-0.044 | 0.3 |
| Tolclofos  -methyl | 2.5 | 0.003-0.072 |  |  |  |  |  |  |  |  |  |  |  |  |  |  |  |  |  |  | 0.3 |
| dimethoate |  |  | 5.4 | 0.004-0.009 |  |  |  |  |  |  |  |  |  |  |  |  |  |  |  |  | 0.2 |
| Metsulfuron  -methyl |  |  | 5.4 | 0.011-0.020 |  |  |  |  |  |  |  |  |  |  |  |  |  |  |  |  | 0.2 |
| fenitrothion |  |  |  |  |  |  |  |  |  |  | 6.7 | 0.003-0.495 |  |  |  |  |  |  |  |  | 0.2 |
| amitraz |  |  | 2.7 | 0.007 |  |  |  |  |  |  |  |  |  |  |  |  |  |  |  |  | 0.1 |
| fenamiphos |  |  |  |  |  |  |  |  |  |  | 3.3 | 0.004-0.008 |  |  |  |  |  |  |  |  | 0.1 |
| fenobucarb |  |  |  |  |  |  |  |  |  |  | 3.3 | 0.020-0.337 |  |  |  |  |  |  |  |  | 0.1 |
