# Supplementary material for: Levels and Health Risk of Pesticide Residues in Chinese Herbal Medicines
Source: Front Pharmacol. 2022 Feb 1;12:818268. doi: 10.3389/fphar.2021.818268 (PMC8844025; doi:10.3389/fphar.2021.818268)
Supplement: Supplementary file 3 [file Table2.doc]

Table 2 Long-term and short-term risk assessment of 10 CHMs

| **Pesticide** | **HQc** | | | | | | | | | | **HQa** | | | | | | | | | |
| --- | --- | --- | --- | --- | --- | --- | --- | --- | --- | --- | --- | --- | --- | --- | --- | --- | --- | --- | --- | --- |
|  | GR | LF | HH | OR | AR | CR | FC | PR | GF | LJ | GR | LF | HH | OR | AR | CR | FC | PR | GF | LJ |
| carbendazim | 0.000081 | 0.001318 | 0.009170 | 0.000039 | 0.000033 | 0.000262 |  | 0.000091 |  |  | 0.000079 | 0.003406 | 0.071525 | 0.000408 | 0.000292 | 0.000488 |  | 0.007176 |  |  |
| chlorpyrifos | 0.000826 | 0.000505 | 0.001249 | 0.000078 | 0.098690 | 0.013696 | 0.000188 | 0.000080 | 0.000296 | 0.001261 | 0.000742 | 0.000586 | 0.008583 | 0.000052 | 0.031250 | 0.026869 | 0.000346 | 0.000101 | 0.000478 | 0.001007 |
| paclobutrazol |  |  | 0.000091 | 0.001156 | 0.000240 | 0.000082 |  | 0.000008 |  |  |  |  |  |  |  |  |  |  |  |  |
| difenoconazole | 0.000654 | 0.000823 | 0.000445 | 0.000075 |  | 0.001939 |  | 0.000074 | 0.000069 | 0.000050 | 0.000188 | 0.000336 | 0.000519 | 0.000020 |  | 0.000657 |  | 0.000026 | 0.000012 | 0.000344 |
| acetamiprid | 0.000148 | 0.000332 | 0.000068 | 0.000029 | 0.000110 | 0.001293 | 0.000039 |  | 0.000025 | 0.000017 | 0.000075 | 0.000434 | 0.000250 | 0.000036 | 0.000111 | 0.002135 | 0.000129 |  | 0.000019 | 0.000039 |
| carbosulfan |  | 0.000559 | 0.000204 | 0.000011 |  | 0.000096 | 0.000019 | 0.000011 | 0.000009 |  |  | 0.001437 | 0.015058 | 0.000013 |  | 0.000726 | 0.000346 | 0.000021 | 0.000090 |  |
| tebuconazole |  | 0.000660 | 0.000169 | 0.000073 | 0.000080 | 0.000871 | 0.000336 | 0.000067 | 0.000136 | 0.000050 |  | 0.004692 | 0.002124 | 0.000063 | 0.000417 | 0.010304 | 0.008333 | 0.000276 | 0.000135 | 0.000268 |
| quintozene |  | 0.003126 | 0.000668 |  |  | 0.003467 |  |  | 0.000677 | 0.006706 |  | 0.014775 | 0.029063 |  |  | 0.017789 |  |  | 0.003146 | 0.009269 |
| imidacloprid |  | 0.001976 | 0.000103 | 0.000012 |  | 0.000064 | 0.000015 | 0.000013 | 0.000012 |  |  | 0.016979 | 0.000598 | 0.000003 |  | 0.000106 | 0.000034 | 0.000016 | 0.000010 |  |
| cypermethrins | 0.078536 |  |  |  |  |  | 0.000088 | 0.000102 |  |  |  |  |  |  |  |  |  |  |  |  |
| pyridaben |  | 0.000449 | 0.001948 |  |  | 0.000241 | 0.000087 |  | 0.000066 | 0.000990 |  |  |  |  |  |  |  |  |  |  |
| thiophanate-  methyl |  | 0.000411 | 0.001304 |  |  | 0.000090 |  |  | 0.000154 | 0.012616 |  | 0.002623 | 0.066250 |  |  | 0.000825 |  |  | 0.002667 | 0.038301 |
| triazophos |  |  | 0.003327 |  | 0.123886 | 0.015588 |  |  | 0.003468 |  |  |  | 0.179167 |  | 1.125000 | 0.238625 |  |  | 0.062917 |  |
| cyhalothrin |  | 0.000061 | 0.000298 |  |  | 0.000834 | 0.000047 |  | 0.000184 | 0.000017 |  | 0.000629 | 0.015417 |  |  | 0.011082 | 0.003194 |  | 0.004139 | 0.000400 |
| procymidone |  | 0.000563 | 0.000283 | 0.000009 |  |  |  | 0.000010 |  |  |  |  |  |  |  |  |  |  |  |  |
| fenpropathrin | 0.000131 | 0.000008 | 0.000112 | 0.000009 | 0.000034 |  | 0.000022 | 0.000008 |  |  | 0.001350 | 0.000029 | 0.007542 | 0.000125 | 0.000500 |  | 0.000958 | 0.000141 |  |  |
| fenvalerate |  | 0.000001 | 0.000001 |  |  |  |  |  |  |  |  |  |  |  |  |  |  |  |  |  |
| propargite |  | 0.001528 | 0.000220 |  |  | 0.000556 |  |  | 0.000040 | 0.002407 |  | 0.001351 | 0.001958 |  |  | 0.002598 |  |  | 0.000031 | 0.001203 |
| chlorantraniliprole |  | 0.004098 | 0.000666 |  |  | 0.004040 |  |  | 0.000072 |  |  |  |  |  |  |  |  |  |  |  |
| iprodione | 0.000094 |  | 0.000026 | 0.000012 | 0.000117 | 0.000075 |  |  |  |  | 0.000232 |  | 0.000111 | 0.000028 | 0.000417 | 0.000590 |  |  |  |  |
| propiconazole | 0.000154 |  |  |  |  |  |  |  |  |  |  |  |  |  |  |  |  |  |  |  |
| phorate | 0.003196 |  | 0.001090 | 0.001093 | 0.002279 | 0.005617 |  | 0.001022 | 0.001055 |  | 0.000972 |  | 0.001550 | 0.000972 | 0.011111 | 0.007028 |  | 0.000417 | 0.000417 |  |
| azoxystrobin | 0.000005 |  | 0.000006 | 0.000008 | 0.000007 |  | 0.000007 | 0.000007 | 0.000004 | 0.000003 | 0.000003 |  | 0.000035 | 0.000235 | 0.000042 |  | 0.000053 | 0.000032 | 0.000002 | 0.000051 |
| permethrin | 0.000019 | 0.000007 | 0.000016 |  |  |  |  |  |  |  |  |  |  |  |  |  |  |  |  |  |
| dimethomorph |  |  | 0.000039 |  |  |  | 0.000026 | 0.000015 |  | 0.000165 |  |  |  |  |  |  |  |  |  |  |
| prochloraz | 0.004375 |  |  |  | 0.001021 | 0.001800 | 0.003115 |  |  |  |  |  |  |  |  |  |  |  |  |  |
| propamocarb hydrochloride |  | 0.000219 | 0.000232 |  |  | 0.000899 |  | 0.000145 |  | 0.000100 |  |  |  |  |  |  |  |  |  |  |
| endosulfan | 0.000048 |  | 0.000051 | 0.000113 |  |  |  | 0.000025 | 0.000025 |  | 0.000100 |  | 0.000662 | 0.002589 |  |  |  | 0.000028 | 0.000037 |  |
| fipronil |  | 0.004611 | 0.013254 |  |  | 0.008113 |  | 0.003750 |  | 0.016299 |  | 0.001569 | 0.072222 |  |  | 0.008889 |  | 0.000738 |  | 0.018613 |
| myclobutanil |  |  |  | 0.000190 |  |  |  |  | 0.000683 | 0.000167 |  |  |  | 0.003333 |  |  |  |  | 0.018833 | 0.000353 |
| phoxim |  | 0.000093 | 0.000098 | 0.000076 | 0.000085 | 0.030180 | 0.000082 | 0.000075 | 0.000074 |  |  | 0.000125 | 0.000303 | 0.000026 | 0.000167 | 0.020072 | 0.000121 | 0.000027 | 0.000013 |  |
| bifenthrin | 0.000002 |  |  |  |  |  |  |  |  |  |  |  |  |  |  |  |  |  |  |  |
| cyprodinil |  |  | 0.000401 |  |  | 0.000598 |  |  |  |  |  |  | 0.039792 |  |  | 0.008394 |  |  |  |  |
| cyfluthrin |  |  |  | 0.000088 |  |  |  |  |  |  |  |  |  |  |  |  |  |  |  |  |
| flusilazole | 0.000049 | 0.000032 | 0.000063 |  | 0.000033 |  | 0.000037 | 0.000026 |  | 0.000017 | 0.000010 | 0.000037 | 0.000416 |  | 0.000083 |  | 0.000064 | 0.000064 |  | 0.000088 |
| hexaconazole |  |  | 0.000041 | 0.000037 |  |  |  | 0.000037 | 0.000036 |  |  |  |  |  |  |  |  |  |  |  |
| etoxazole |  |  | 0.000415 |  |  |  |  |  | 0.000096 | 0.000050 |  |  | 0.037500 |  |  |  |  |  | 0.001396 | 0.003266 |
| hexaflumuron |  |  | 0.000161 |  |  | 0.003163 |  |  | 0.000025 | 0.000017 |  |  | 0.000167 |  |  | 0.000975 |  |  | 0.000003 | 0.000003 |
| diflubenzuron | 0.000163 |  |  |  |  |  |  |  |  |  |  |  |  |  |  |  |  |  |  |  |
| methidathion | 0.000278 | 0.000112 |  |  |  |  |  | 0.000107 |  | 0.000071 | 0.000396 | 0.000217 |  |  |  |  |  | 0.000125 |  | 0.000332 |
| omethoate |  |  | 0.000019 |  |  | 0.000154 |  | 0.000015 | 0.000015 |  |  |  |  |  |  |  |  |  |  |  |
| etofenprox |  |  | 0.000103 |  |  |  | 0.000039 |  | 0.000066 |  |  |  |  |  |  |  |  |  |  |  |
| pyraclostrobin |  | 0.000041 |  |  |  | 0.000233 |  |  |  |  |  |  |  |  |  |  |  |  |  |  |
| Imazalil |  |  |  |  |  | 0.020129 |  |  |  |  |  |  |  |  |  | 0.018650 |  |  |  |  |
| pyrimethanil |  |  | 0.000209 |  |  | 0.000285 |  |  |  |  |  |  | 0.000258 |  |  | 0.000033 |  |  |  |  |
| metalaxyl |  |  |  |  |  |  |  |  |  | 0.001667 |  |  |  |  |  |  |  |  |  |  |
| bitertanol |  |  |  |  |  |  | 0.000031 |  |  |  |  |  |  |  |  |  | 0.000008 |  |  |  |
| Isofenphos  -methyl |  |  | 0.002533 |  |  | 0.000429 |  |  |  |  |  |  |  |  |  |  |  |  |  |  |
| BHC |  |  | 0.029300 |  |  | 0.016163 |  |  |  |  |  |  |  |  |  |  |  |  |  |  |
| butralin |  |  |  |  |  | 0.001201 |  |  |  |  |  |  |  |  |  | 0.008984 |  |  |  |  |
| parathion |  |  |  | 0.001177 |  |  |  |  | 0.001143 |  |  |  |  | 0.000625 |  |  |  |  | 0.000625 |  |
| triadimefon | 0.000016 |  | 0.000004 |  |  |  |  | 0.000004 |  |  |  |  |  |  |  |  |  |  |  |  |
| profenofos | 0.000040 |  | 0.000013 |  | 0.000011 |  | 0.000012 | 0.000009 |  |  |  |  |  |  |  |  |  |  |  |  |
| isazofos | 0.000300 |  | 0.000187 |  |  |  |  | 0.000186 |  |  |  |  |  |  |  |  |  |  |  |  |
| DDT |  |  |  |  |  | 0.000843 | 0.000882 |  |  | 0.000500 |  |  |  |  |  |  |  |  |  |  |
| trifluralin | 0.003396 |  |  |  |  |  | 0.000205 |  |  |  |  |  |  |  |  |  |  |  |  |  |
| carbaryl |  |  |  |  |  |  |  |  |  | 0.000003 |  |  |  |  |  |  |  |  |  | 0.000106 |
| chlorfenapyr |  |  |  |  | 0.000924 |  |  |  |  | 0.000125 |  |  |  |  | 0.013333 |  |  |  |  | 0.002877 |
| 2,4-D butylate |  |  | 0.000052 |  |  |  |  | 0.000025 |  | 0.000017 |  |  | 0.000429 |  |  |  |  | 0.000020 |  | 0.000141 |
| cadusafos |  | 0.000083 | 0.000078 |  |  | 0.000172 |  |  | 0.000074 |  |  | 0.000546 | 0.000409 |  |  | 0.001988 |  |  | 0.000125 |  |
| acetochlor | 0.000349 | 0.000094 |  |  |  | 0.001137 |  |  |  |  |  |  |  |  |  |  |  |  |  |  |
| methamidophos |  |  | 0.000004 | 0.000004 |  | 0.000005 |  |  |  |  |  |  |  |  |  |  |  |  |  |  |
| dicofol |  |  |  | 0.000079 |  |  | 0.000076 |  | 0.000300 |  |  |  |  |  |  |  |  |  |  |  |
| isocarbophos |  |  |  |  |  |  |  |  |  | 0.000020 |  |  |  |  |  |  |  |  |  |  |
| fenitrothion |  |  | 0.000093 |  |  |  |  |  |  | 0.000063 |  |  | 0.000006 |  |  |  |  |  |  | 0.000103 |
| coumaphos |  |  |  |  |  |  |  |  |  | 0.000017 |  |  |  |  |  |  |  |  |  | 0.001416 |
| propoxur |  |  |  |  |  | 0.020533 |  |  |  |  |  |  |  |  |  |  |  |  |  |  |
| buprofezin |  |  |  |  |  | 0.000019 |  |  |  |  |  |  |  |  |  | 0.000078 |  |  |  |  |
| terbufos |  |  |  |  |  | 0.003416 |  |  |  | 0.000250 |  |  |  |  |  | 0.001483 |  |  |  | 0.000014 |
| fenamiphos |  |  | 0.000028 | 0.000018 |  |  |  | 0.000018 |  |  |  |  |  |  |  |  |  |  |  |  |
| dimethoate | 0.000010 |  |  |  |  |  |  |  |  |  |  |  |  |  |  |  |  |  |  |  |
| dichlorvos |  |  |  |  |  | 0.001683 |  |  |  |  |  |  |  |  |  | 0.003417 |  |  |  |  |
| amitraz |  |  |  |  |  | 0.003283 |  |  | 0.002511 |  |  |  |  |  |  |  |  |  |  |  |
| fenpyroximate |  |  | 0.000170 |  |  |  | 0.000081 | 0.000112 |  | 0.000050 |  |  | 0.000029 |  |  |  | 0.000011 | 0.000078 |  | 0.000017 |
| diethofencarb |  |  |  |  |  |  |  |  |  | 0.000125 |  |  |  |  |  |  |  |  |  | 0.001658 |
| fludioxonil |  |  |  |  |  |  |  |  | 0.000185 | 0.000125 |  |  |  |  |  |  |  |  | 0.000013 | 0.001607 |
| Tolclofos  -methyl |  |  |  |  |  |  |  |  |  | 0.000017 |  |  |  |  |  |  |  |  |  | 0.000183 |
| Metsulfuron  -methyl | 0.000161 |  |  |  |  |  |  |  |  |  |  |  |  |  |  |  |  |  |  |  |
| prometryn |  | 0.000340 |  |  |  |  |  |  |  |  |  | 0.000188 |  |  |  |  |  |  |  |  |
| piperonyl butoxide |  | 0.000003 |  |  |  |  |  |  |  |  |  |  |  |  |  |  |  |  |  |  |
| uniconazole |  |  |  |  | 0.000790 | 0.000765 |  |  |  |  |  |  |  |  | 0.000208 | 0.000045 |  |  |  |  |
| malathion |  | 0.000078 |  |  |  |  |  |  |  |  |  | 0.000279 |  |  |  |  |  |  |  |  |
| fenobucarb |  |  |  |  |  | 0.000986 |  |  |  |  |  |  |  |  |  | 0.001069 |  |  |  |  |
| HI | 0.093 | 0.022 | 0.069 | 0.004 | 0.228 | 0.165 | 0.005 | 0.006 | 0.012 | 0.044 |  |  |  |  |  |  |  |  |  |  |
